# Genomic Characterization of a Wild-Type *Bovine alphaherpesvirus 1* (BoAHV-1) Strain Isolated in an Outbreak in Central Italy

**DOI:** 10.3390/v16010150

**Published:** 2024-01-19

**Authors:** Stefano Petrini, Valentina Curini, Cecilia Righi, Cesare Cammà, Valeria Di Lollo, Elena Tinelli, Luana Fiorella Mincarelli, Elisabetta Rossi, Giulia Costantino, Barbara Secondini, Silvia Pirani, Monica Giammarioli, Francesco Feliziani

**Affiliations:** 1 National Reference Centre for Infectious Bovine Rhinotracheitis (IBR), Istituto Zooprofilattico Sperimentale Umbria-Marche “Togo Rosati”, 06126 Perugia, Italy; s.petrini@izsum.it (S.P.); e.tinelli@izsum.it (E.T.); e.rossi@izsum.it (E.R.); g.costantino@izsum.it (G.C.); s.pirani@izsum.it (S.P.); m.giammarioli@izsum.it (M.G.); f.feliziani@izsum.it (F.F.); 2 National Reference Center for Whole Genome Sequencing of Microbial Pathogens, Istituto Zooprofilattico Sperimentale Abruzzo-Molise “G. Caporale”, 64100 Teramo, Italy; v.curini@izs.it (V.C.); c.camma@izs.it (C.C.); v.dilollo@izs.it (V.D.L.); l.mincarelli@izs.it (L.F.M.); b.secondini@izs.it (B.S.)

**Keywords:** wild-type bovine alphaherpesvirus-1, next-generation sequencing, whole-genome sequencing

## Abstract

*Bovine alphaherpesvirus-1* (BoAHV-1) infection is common in cattle worldwide. However, information on the spread of BoAHV-1-circulating strains in Italy remains limited. In this study, we investigated an outbreak characterized by severe respiratory symptoms in a cattle herd (*n* = 30) located in Central Italy. BoAHV-1 was isolated from three cattle in a cell culture, which confirmed viral infection. Next, we characterized one (16453/07 TN) of the three isolates of BoAHV-1 using whole-genome sequencing. BLASTn and phylogenetic analysis revealed a nucleotide identity >99% with all BoAHV-1 strains belonging to subtype 1.1, highlighting the genetic stability of the virus. This study reports the first full genomic characterization of a BoAHV-1 isolate in Italy, enriching our understanding of the genetic characteristics of the circulating BoAHV-1 strain in Italy.

## 1. Introduction

*Bovine alphaherpesvirus-1* (BoAHV-1) is a member of the *Herpesviridae* family, subfamily *Alphaherpesvirinae*, and genus *Varicellovirus* [1] which causes different clinical syndromes in cattle and is referred to as infectious bovine rhinotracheitis (IBR). Additionally, it leads to reproductive disorders, including abortion and infertility [2,3]. BoAHV-1 infection causes severe economic losses in the livestock industry worldwide. Thus, the European Union (EU) has implemented regulations concerning the movement of animals within its member states [4,5] and the mandatory reporting of positive cases in areas designated as IBR-free [6].

In 1954, the first report of respiratory disease caused by BoAHV-1 was reported in Los Angeles (CA, USA) [7,8]. Subsequently, in 1955–1956, BoAHV-1 was isolated in cell culture from nasal samples collected from experimentally infected cattle in Southern California (Los Angeles strain) and from cattle located in Colorado (Cooper strain) [9,10]. In Italy, BoAHV-1 was first reported by Prof. Bruno Moretti at the University of Perugia in 1964 [11]. Since then, the virus has spread worldwide, and to date, three genetic subtypes (1.1, 1.2a, and 1.2b) have been identified [12]. Furthermore, a list of reference genomic sequences of BoAHV-1 has also been published [12,13,14,15,16,17].

BoAHV-1 infections in cattle are relatively common in Italy, as evidenced by a herd seroprevalence of 30.6% in 2019 [18,19]. The herd prevalence rates vary regionally, with higher estimates in South (64.3%) and Central Italy (43.4%) than in North Italy (25.3%) [18]. However, despite the prevalence of infections, the genetic features of the virus are poorly understood within the Italian context.

An IBR outbreak was reported in March 2007 in a beef cattle herd located in Central Italy (Marche Region), which is currently an integral part of the Biobank of the National Reference Center for Infectious Bovine Rhinotracheitis at Istituto Zooprofilattico Sperimentale Umbria-Marche “Togo Rosati”, Perugia, Italy. The herd comprised nine-to-twelve-month-old Charolais cattle (*n* = 30) originally from France. One week after they arrived at the farm, 12 animals exhibited severe respiratory symptoms, including fever, nasal mucopurulent exudate, dyspnea, and cough. Anamnesis revealed that these animals previously resided in an IBR-free farm located in a non-IBR-free area. Furthermore, the animals had not been vaccinated against the main respiratory diseases in cattle. We hypothesized that these animals might be infected with a wild-type strain of BoAHV-1. Subsequently, we isolated and identified a strain of BoAHV-1 (unpublished data), which has been used in several experimental challenge infections in calves and water buffaloes [20,21]. In this study, we aimed to characterize the genome of the virus responsible for the respiratory outbreaks in 2007. The findings of this study could help us to develop eradication strategies for IBR, particularly in areas where control/eradication plans are not active, as indicated by recent European regulations [4,5,6,22,23].

## 2. Materials and Methods

Blood samples and nasal swabs were collected from the 12 animals exhibiting severe respiratory symptoms, transported to the laboratory under refrigeration within 2 h of collection, and tested for different pathogens. Specifically, *Bovine alphaherpesvirus 1* (BoAHV-1), *Bovine respiratory syncytial virus* (BRSV), *Bovine parainfluenza-3* (BPI-3) *virus*, *Bovine viral diarrhea virus* (BVDV), *Mannheimia hemolytica*, *Pasteurella multocida*, *Histophilus somni*, and *Mycoplasma bovis* were evaluated.

The blood samples were centrifuged (Centrifuge 5810 R, Eppendorf^®^, Milan, Italy) at 1250× *g* for 10 min at 4 °C, and the serum samples obtained were tested using different commercial enzyme-linked immunosorbent assays (ELISAs) and virus neutralization (VN) assays. In particular, IDEXX IBR gB X3 Ab (IDEXX, Westbrook, ME, USA), PRIMACHECK BRSV Ab (Agrolabo S.p.A., Torino, Italy), PRIMACHECK PI-3 Ab (Agrolabo S.p.A. Torino, Italy), and IDEXX BVDV p80 Ab (Westbrook, ME, USA) were used for the evaluation of serum samples following the protocols available with respective ELISA kits. VN assays against BoAHV-1 were performed according to the World Organisation for Animal Health’s (WOAH, founded as OIE) *Manual of Diagnostic Tests and Vaccines for Terrestrial Animals* [24].

Nasal swab samples were collected in duplicate using sterile transport swabs (MicroBiotech S.r.l., Lecce, Italy; Citoswab, transport swab, Haimen, China). Bacteriological investigations were conducted using the protocols previously described by Carter and Coll [25]. For virological investigations, the nasal swabs were collected and transported in Minimum Essential Medium (MEM; Euroclone, Milan, Italy) supplemented with 5× antibiotics/antifungals (5000 U.I. Penicillin, 2500 U.I. Streptomycin, and 25 µg amphotericin B (Sigma-Aldrich, Milan, Italy). The MEM was filtered using a 0.22 μm filter (Sartorius Stedim Biotech Gmbh, Goettingen, Germany). Subsequently, 0.1 mL of each sample was added to 24-well plates (NuncTM, Thermo Scientific, Milan, Italy) containing Madin–Darby Bovine Kidney (MDBK) cell cultures with 10% fetal bovine serum (Euroclone S.p.A, Milan, Italy). The cells were provided by the Biobanking of Veterinary Resources (BVR, Brescia, Italy) and identified using the code BS CL 63. The plates were incubated at 37 °C in a 5% CO_2_ atmosphere for 7 days and were checked daily for the presence of a cytopathic effect (CPE) induced by BoAHV-1. Positive samples were confirmed using gB real-time PCR for the glycoprotein B (gB) gene of BoAHV-1 [24].

Subsequently, strain 16453/07 TN of BoAHV-1 was selected and used to infect MDBK cells. The infected cells were propagated in a cell culture flask of 75 cm^2^ (Corning, NY, USA). Uninfected MDBK cells were grown in a separate cell culture flask (Corning) of the same size and used as a negative control.

The virus was seeded 1 × 10^2.00^ TCID_50_/mL in MEM and incubated for 1 h at 37 °C in a 5% CO_2_ atmosphere, followed by the addition of MEM plus 5% FBS. CPE was evaluated daily using an inverted microscope (Olympus IX51; Olympus, Milan, Italy). The flasks exhibiting 80–90% CPE were frozen at −80 °C. Subsequently, the contents of cell flasks were centrifuged (Centrifuge 5810 R) at 1250× *g* for 10 min at 4 °C following three freezing/thawing cycles.

The supernatant was collected and ultracentrifuged (Beckman Coulter, Inc., Indianapolis, CA, USA) at 16,000 rpm for 1.5 h at 4 °C in a Beckman 45 Ti rotor with a polycarbonate centrifuge bottle (Beckman Coulter, cat # 355654). The obtained pellet was suspended in 1 mL of MEM with 1× antibiotics (5000 U.I. Penicillin, 2500 U.I. Streptomycin, and 25 µg amphotericin B; Sigma-Aldrich) and stored at −80 °C.

DNA was extracted as described by Petrini et al. [21] and processed at the National Reference Center for Whole Genome Sequencing of Microbial Pathogens (GENPAT, Teramo, Italy) using an Illumina DNA Prep kit (Illumina Inc., San Diego, CA, USA). Sequencing was performed on Illumina’s next-generation sequencing NexSeq2000 platform (Illumina Inc.) using the NextSeq 1000/2000 P2 Reagent kit (300 cycles, 150 bp paired-end reads). After evaluating the quality of raw reads using FastQC v0.11.5 and trimming using Trimmomatic v0.36, the quality-filtered reads were depleted on the GENPAT platform (https://genpat.izs.it/cmdbuild/ui/#login, accessed on 26 April 2023). Subsequently, trimmed reads were mapped to the reference BoAHV-1 Cooper strain genome (GenBank Accession JX898220) using Snippy (version 4.5.1) (https://github.com/tseemann/snippy, accessed on 26 April 2023), and the consensus sequence was generated using iVar (version 1.3). Finally, the obtained consensus sequence and 51 complete BoAHV-1 genomes (including those reported mainly from the USA along with those from China, India, and Australia) available in NCBI (https://www.ncbi.nlm.nih.gov/nucleotide/, accessed on 5 June 2023 Month Year) were used for phylogenetic analysis using Version 11 of Molecular Evolutionary Genetics Analysis (MEGA) software (https://www.megasoftware.net/, accessed on 8 June 2023).

## 3. Results

The ELISA results showed that all 12 serum samples tested positive for BRSV, whereas 11 of the 12 samples tested positive for BPI-3. No seropositivity was detected by BVDV ELISA. Neutralizing antibodies (NAs) against BoAHV-1 showed titers ranging from 1:2 to 1:256 and were recorded in only nine animals. The remaining three cattle tested negative for NAs. After 48–72 h of seeding, virological investigations revealed that 3 of the 12 samples exhibited shrunk or detached round and enlarged cells speculated as a herpesvirus. Subsequently, the virus was identified as BoAHV-1 using gB real-time PCR. In addition, bacteriological investigations identified four *Mycoplasma bovis*-positive samples (Table 1).

**Figure 1:**
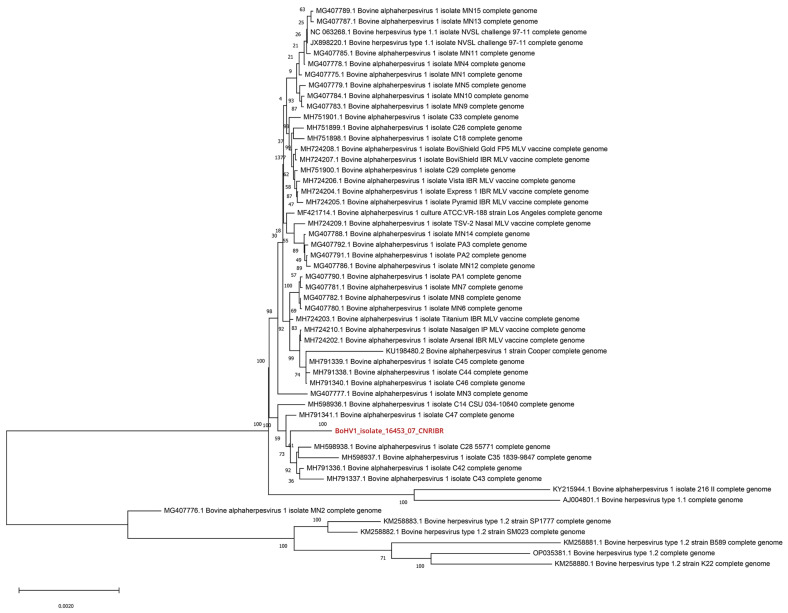
Phylogenetic analysis of the strain 16453/07 TN of BoAHV-1 performed using the maximum likelihood method and the Tamura 3-parameter model, with 100 bootstrap replicates, including 51 nucleotide sequences available in NCBI.

Approximately 8 million raw reads were obtained by whole-genome sequencing of BoAHV-1 (isolate 16453/07 TN; corresponding to sample Id.4 in Table 1), and after quality control and trimming, 7,436,870 reads were retained. Mapping of these reads with the reference BoAHV-1 Cooper strain genome produced a consensus sequence of 134,821 bp with an average vertical coverage of 1058 × and horizontal coverage of 99.5%. The complete genomic sequence of the isolate has been deposited in GenBank (accession number: OR211605). The BLAST analysis revealed a nucleotide identity >99% with all complete type 1.1 BoAHV-1 genomes. Furthermore, subsequent phylogenetic analysis using the complete BoAHV-1 genomes available in the NCBI database located the isolated strain among BoAHV-1 subtype 1.1, along with strains identified from the USA (Figure 1). Single-nucleotide polymorphism (SNP) analysis revealed a profile closely related to field isolates described to date [15,17,26] and dissimilar to the SNP pattern associated with vaccine viruses [15,17,26]. The strain 16453/07 TN of BoAHV-1 shared 23 unique SNPs (Table 2; denoted using asterisks) with genomes of reported non-vaccine-associated wild-type viruses [15,17]. In particular, the isolate characterized in this study showed an SNP profile more similar to that observed by D’Offay et al. [15] in the respiratory samples in comparision to the profile observedin the fetal samples.

## 4. Discussion

BoAHV-1 infection is considered one of the main causes of economic losses in the livestock industry worldwide and is associated with animal morbidity and restrictions on movement and trade. Currently, the Delegated Regulation (EU) 2020/689 [6] establishes the ground rules for surveillance programs, disease control strategies, and measures to be applied in cases of suspected and confirmed infections. Therefore, it is important to quickly identify and report on the circulation of IBR for granting and maintaining IBR-free zones in regions with IBR-free farms.

It is necessary to keep in mind that the virus was isolated in 2007 from sick beef cattle before IBR surveillance or eradication programs had been established in the Marche Region. Therefore, we speculate that BoAHV-1 infection may have arrived at the farm from the import of latently infected French animals, triggering an IBR outbreak. Subsequent epidemiological investigations did not demonstrate the spread of the virus to nearby farms because the infected farm was located in a mountainous area. Moreover, the nearest farms were more than 5 km from the outbreak site. These results are similar to those of Li et al. [26], who showed that 5.1 km is the distance from the outbreak where clinical cases of malignant catarrhal fever (FCM) caused by ovine herpesvirus 2 (OvHV-2) can still be observed. Together, these results suggest that the environmental situation and distance of the other farms affected by the outbreak must have influenced the reduction in viral spread.

Furthermore, the NA titers were 1:4 in all three samples from which BoAHV-1 was isolated and confirmed by gB real-time PCR. The remaining animals showed titers ranging from 1:2 to 1:256 in the absence of virus isolation. These results highlight viral circulation at different stages of infection within the host population. An IBR disease outbreak involves a complex interaction between the virus and the host immune response. In particular, a low NA titer associated with viral excretion may indicate the end of the viremic phase and the beginning of the immune phase. These results are similar to those of previous studies on calves and water buffaloes challenge-infected with BoAHV-1 and Bubaline alphaherpesvirus 1, respectively [21,27]. In contrast, NA levels were increased up to 1:256 in the absence of viral excretion. These results are consistent with those reported by other authors [20,28] and can be interpreted as an active immune response to viremia.

The strain 16453/07 TN of BoAHV-1 has been used for challenge infections in calves and water buffaloes for the evaluation of the safety and efficacy of different marker vaccines. Additionally, the virus has been used in various studies to evaluate vaccine latency [20,21]. In all challenge infections with the BoAHV-1 strain 16453/07 TN, the animals exhibited nasal mucopurulent exudate, dyspnea, and cough. In addition, lesions consisting of pseudomembranes were observed in the nasal mucosa. The rectal temperatures of the challenge-infected animals increased up to 41.0–41.5 °C until post-challenge day (PCD) 8. Furthermore, the virus was excreted on PCD 2 with a high titer (10^6.24^ TCID_50_/mL) [20] and was detected at 21.00 Ct using gB real-time PCR [21]. These results demonstrate that Koch’s postulates are valid. The virus responsible for the bovine respiratory syndrome was (i) isolated from cell cultures of sick animals, (ii) propagated on cell cultures, (iii) injected into different animals showing infectious bovine rhinotracheitis (IBR), and (iv) re-isolated from diseased animals [29].

In the present study, the complete genome sequence of BoAHV-1 (OR211605) was obtained from the 16453/07 TN isolate. BLAST analysis of the obtained sequence showed a nucleotide identity >99% with all complete type 1.1 BoAHV-1 genomes available in NCBI, as highlighted by phylogenetic analysis. SNP analysis demonstrated an association between variant profiles of BoAHV-1 16453_07 TN isolates and field samples sequenced and a difference from those of vaccine viruses and isolates from cases associated with vaccination [15,17,26]. A high nucleotide identity and similar SNP profile to samples isolated in other countries in the past few years confirm that BoAHV-1.1 is a genetically stable virus, although there are marked differences in the temporal, geographical, and disease-related origins of these wild-type viruses [12].

Routine studies and comparisons of viral genomes play an important role in determining the occurrence of recombination phenomena, which can lead to the creation of new viral forms with specific pathogenic characteristics [30]. Recombination is a mechanism of genetic variation in herpesviruses [31] that occurs during co-infection or during an infection that is delayed in a short interval of time. However, this type of recombination is not well known in nature. Several studies have shown that recombination can occur when two viral strains of the same herpesvirus species are inoculated [32,33]. Furthermore, herpesvirus recombination has been detected after primary infection or reactivation of latent viruses [34]. Several studies reported in China demonstrated the genetic recombination of different pseudorabies viruses, suggesting a zoonotic role by inducing eye disease, encephalitis, and endophthalmitis [35].

To our knowledge, this is the first study reporting the complete genome sequence of the causative agent of IBR in Italy. Further studies are needed to detect viral structural variants associated with BoAHV-1 that can influence the virulence, spread, and dynamics of infection.

## 5. Conclusions

In this study, we isolated and sequenced the complete genome of a BoAHV-1 strain isolated in 2007 from the Istituto Zooprofilattico Sperimentale Umbria-Marche, Perugia, Italy. The virus was isolated from the nasal swabs of cattle with respiratory diseases. Whole-genome sequencing results show that the virus belonged to BoAHV-1 subtype 1.1.

## Figures and Tables

**Table 1:** Serological and virological results of serum samples obtained from Charolais cattle with severe respiratory symptoms (*n* = 12).

SAMPLES Id.	IBR			BVDV	BPI−3	BRSV	EBS ^
	ELISA ^1^	VN	VIRAL ISOLATION	ELISA ^2^	ELISA ^3^	ELISA ^4^	
**1**	**−**	**4**	**+**	**−**	***−***	**+**	**+**
**2**	**+**	**4**	**+**	**−**	**+**	**+**	**+**
**3**	**+**	**−**	**−**	**−**	**+**	**+**	**+**
**4**	**+**	**4**	**+**	**−**	**+**	**+**	**+**
**5**	**+**	**64**	**−**	**−**	**+**	**+**	**−**
**6**	**−**	**2**	**−**	**−**	**+**	**+**	**−**
**7**	**−**	**8**	**−**	**−**	**+**	**+**	**−**
**8**	**−**	**−**	**−**	**−**	**+**	**+**	**−**
**9**	**+**	**−**	**−**	**−**	**+**	**+**	**−**
**10**	**+**	**256**	**−**	**−**	**+**	**+**	**−**
**11**	**+**	**256**	**−**	**−**	**+**	**+**	**−**
**12**	**+**	**32**	**−**	**−**	**+**	**+**	**−**

ELISA, enzyme-linked immunosorbent assays; ^1^ IDEXX IBR gB X3 Ab (IDEXX, Westbrook, ME, USA); ^2^ IDEXX BVDV p80 Ab (Westbrook, ME, USA); ^3^ PRIMACHECK PI-3 Ab (Agrolabo S.p.A. Torino, Italy); ^4^ PRIMACHECK BRSV Ab (Agrolabo S.p.A., Torino, Italy); IBR, Infectious Bovine Rhinotracheitis; BVDV, Bovine Viral Diarrhea Virus; BPI-3, Bovine Parainfluenza-3; BRSV, Bovine Respiratory Syncytial Virus; VN, virus neutralization titer; ^ positive for *Mycoplasma bovis*.

**Table 2:** Summary of single-nucleotide polymorphisms, insertions, and deletions detected in the genome of BoAHV-1 16453/07 TN isolate.

BoAHV-1 Genome GenBank JX898220 ^a^			BoAHV1_Isolate_16453_07_CRNIBR	
Genes	Position ^b^	Base	Substitutions/Indel	Substitutions/Indel Function
UL53	3466	T	C *	missense_variant
UL52	4560	C	T *	synonymous_variant
UL52	5282	G	T *	missense_variant
UL52	5585	C	T	missense_variant
UL52	6096	A	G	synonymous_variant
Intergenic	10,263	C	T	intergenic_region
UL48	11,591	G	A *	missense_variant
Intergenic	14,295	A	G *	intergenic_region
Intergenic	14,298	A	G *	intergenic_region
UL46	14,409	G	A *	missense_variant
UL44	17,978	G	A	synonymous_variant
UL42	20,390	A	G	missense_variant
UL41	21,814	G	A	synonymous_variant
UL39	23,908	C	G *	synonymous_variant
UL39	25,768	C	T *	synonymous_variant
UL37	28,401	C	T	missense_variant
UL36	31,891	C	T	missense_variant
UL36	32,339	C	T *	synonymous_variant
UL36	34,165	C	G	missense_variant
UL36	34,563	G	A	missense_variant
UL36	38,831	G	A	synonymous_variant
UL36	38,851	C	T	missense_variant
UL36	38,872	C	T	missense_variant
UL36	38,873	A	G	missense_variant
UL36	38,893	C	T	missense_variant
UL36	39,011	A	G	synonymous_variant
UL36	39,052	C	T	missense_variant
UL36	39,118	T	TGACTCCGGCCCC	conservative_inframe_insertion
UL36	40,190	G	A	synonymous_variant
UL35	41,010	C	T	missense_variant
UL32	43,445	T	C	synonymous_variant
Intergenic	44,548	T	TGCTCTC	intergenic_region
UL31	45,722	G	T	synonymous_variant
UL30	46,420	C	G	missense_variant
UL30	46,433	C	T	synonymous_variant
UL30	46,441	G	C	missense_variant
UL30	48,353	C	T	synonymous_variant
UL29	50,436	C	T	synonymous_variant
UL29	50,601	T	C	synonymous_variant
UL29	52,827	C	T	synonymous_variant
Intergenic	53,199	AGG	A	intergenic_region
UL22	64,824	G	A	synonymous_variant
Intergenic	67,242	T	C	intergenic_region
UL21	68,148	G	T	synonymous_variant
Intergenic	69,860	C	T	intergenic_region
Intergenic	69,914	A	G *	intergenic_region
UL19	73,389	G	A	missense_variant
UL17	76,554	G	A	synonymous_variant
UL14	81,391	C	T	synonymous_variant
UL13	82,423	A	G *	missense_variant
UL13	82,547	A	G *	synonymous_variant
UL13	82,850	G	A	synonymous_variant
UL13	82,894	C	T *	missense_variant
UL12	83,734	C	T	synonymous_variant
UL12	84,022	C	T	synonymous_variant
Intergenic	84,765	CGG	C	intergenic_region
UL9	87,138	C	T	synonymous_variant
Intergenic	88,668	TCCC	T	intergenic_region
UL8	89,828	T	G	missense_variant
Intergenic	90,958	A	G	intergenic_region
UL7	91,609	C	T	missense_variant
UL6	91,928	CGCGGCTGCGGCT	C	conservative_inframe_deletion
UL7	91,976	CGCGGCT	C	conservative_inframe_deletion
UL8	92,740	C	G	missense_variant
UL5	94,599	C	T *	synonymous_variant
UL6	95,871	T	C *	synonymous_variant
Intergenic	98,152	A	G	intergenic_region
Intergenic	98,173	G	A *	intergenic_region
Intergenic	98,175	G	C *	intergenic_region
Intergenic	98,177	T	C *	intergenic_region
UL2	98,640	T	C	synonymous_variant
Intergenic	99,062	AA	GG	intergenic_region
UL0.5	100,236	C	T *	missense_variant
Intergenic	100,678	G	A	intergenic_region
LR-ORF1	101,379	G	C	missense_variant
LR-ORF2	101,405	C	A	synonymous_variant
Intergenic	103,142	GC	G	intergenic_region
US3	116,037	A	G	missense_variant
US3	116,042	A	G	missense_variant
US3	116,043	A	G *	missense_variant
US3	116,045	A	G *	missense_variant
US3	116,092	A	AGAGCGAAAGCGG	conservative_inframe_insertion
US3	116,924	C	T	synonymous_variant
US6	119,361	C	T	synonymous_variant
Intergenic	120,109	C	T	intergenic_region
US7	120,936	A	G *	missense_variant
Intergenic	123,367	GC	G	intergenic_region
US9	123,507	G	A	missense_variant

^a^ The reference genomic map is based on the complete BoAHV-1.1 Cooper genome (GenBank Accession JX898220). ^b^ Nucleotide position on the reference BoAHV-1 Cooper reference genome. * Nucleotides with an asterisk denote SNPs that are present in all BoAHV-1 wild-type viruses [15].

## Data Availability

In this study, all data analyzed were collected as part of routine diagnosis; therefore, according to national legislation, ethical approval and written informed consent were not required.

## References

[1] ICTV 9th Report (2011): Herpesviridae. https://talk.ictvonline.org/ictv-reports/ictv_9th_report/dsdnaviruses-2011/w/dsdnaviruses/91/.

[2] Nandi S., Kumar M., Manohar M., Chauhan S. (2009). Bovine herpes virus infections in cattle. Anim. Health Res. Rev..

[3] Righi C., Franzoni G., Feliziani F., Jones C., Petrini S. (2023). The Cell-Mediated Immune Response against Bovine alphaherpesvirus 1 (BoHV-1) Infection and Vaccination. Vaccines.

[4] (2016). Regulation (EU) 2016/429 of the European Parliament and of the Council of 9 March 2016, on Transmissible Animal Diseases and Amending and Repealing Certain Acts in the Area of Animal Health (Animal Health Law). Off. J. Eur. Union.

[5] (2020). Commission Delegated Regulation (EU) 2020/688 of 17 December 2019 Supplementing Regulation (EU) 2016/429 of the European Parliament and of the Council, as Regards Animal Health Requirements for Movements within the Union of Terrestrial Animals and Hatching Eggs.

[6] (2020). Commission Delegated Regulation (EU) 2020/689 of 17 December 2019 Supplementing Regulation (EU) 2016/429 of the European Parliament and of the Council as Regards Rules for Surveillance, Eradication Programmes, and Disease-Free Status for Certain Listed and Emerging Diseases.

[7] Schroeder R.J., Moys M.D. (1954). An acute upper respiratory infection of dairy cattle. J. Am. Vet. Med. Assoc..

[8] McKercher D.G., Moulton J.E., Kendrick J.W., Saito J. Recent developments on upper respiratory disease of cattle. Proceedings of the 59th Annual Meeting US Livestock Sanit.

[9] Miller N.J. (1955). Infectious necrotic rhinotracheitis in cattle. J. Am. Vet. Med. Assoc..

[10] Madin S.H., York C.S., McKercher D.G. (1956). Isolation of the infectious bovine rhinotracheitis virus. Science.

[11] Moretti B., Orfei Z., Mondino G., Persechino A. Isolamento del virus della rinotracheite infettiva del bovino (IBR) in Italia. Proceedings of the Italian Society of Microbiology (TUEMA).

[12] D’Offay J., Fulton W., Fishbein M., Erbele M., Dubovi E. (2019). Isolation of a naturally occurring vaccine/wild-type recombinant bovine herpesvirus type 1 (BoHV-1) from an aborted bovine fetus. Vaccine.

[13] Guo W., Xie J., Liu J., Chen H., Jung Y.S. (2022). The full-genome characterization and phylogenetic analysis of bovine herpesvirus type 1.2 isolated in China. Front. Microbiol..

[14] Dagalp S.B., Farzani F.T., Dogan F., Alkan F., Ozkul A. (2020). Molecular and antigenic characterization of bovine herpesvirus type 1 (BoHV-1) strains from cattle with diverse clinical cases in Turkey. Trop. Anim. Health Prod..

[15] D’Offay J., Fulton W., Eberle R., Dobovi E., Chase C. (2019). Complete genome sequence of bovine herpesvirus type 1.1 (BoHV-1.1) Los Angeles (LA) strain and its genotypic relationship to BoHV-1.1 Cooper and more recently isolated wild type field strains. Arch. Virol..

[16] Chothe S.K., Sebastian A., Thomas A., Nissly R.H., Wolfgang D., Byukusenge M., Mor S.K., Goyal S.M., Albert I., Tewari D. (2018). Whole-genome sequence analysis reveals unique SNP profiles to distinguish vaccine and wild-type strains of bovine herpesvirus-1 (BoHV-1). Virology.

[17] Fulton R.W., D’Offay J.M., Eberle R. (2013). Bovine herpesvirus-1: Comparison and differentiation of vaccine and field strains based on genomic sequence variation. Vaccine.

[18] Tamba M., Pallante I., Petrini S., Feliziani F., Iscaro C., Arrigoni N., Di Sabatino D., Barberio A., Cibin V., Santi A. (2021). Overview of Control Programs for Twenty-Four Infectious Cattle Diseases in Italy. Front. Vet. Sci..

[19] Maresca C., Scoccia E., Dettori A., Felici A., Guarcini R., Petrini S., Quaglia A., Filippini G. (2018). National surveillance plan for infectious bovine rhinotracheitis (IBR) in autochthonous Italian cattle breeds: Results of first year of activity. Vet. Microbiol..

[20] Petrini S., Martucciello A., Grandoni F., De Matteis G., Cappelli G., Giammarioli M., Scoccia E., Grassi C., Righi C., Fusco G. (2021). Evaluation of Safety and Efficacy of an Inactivated Marker Vaccine against Bovine alphaherpesvirus 1 (BoHV-1) in Water Buffalo (*Bubalus bubalis*). Vaccines.

[21] Petrini S., Martucciello A., Righi C., Capelli G., Torresi C., Grassi C., Scoccia E., Costantino G., Casciari C., Sabato R. (2022). Assessment of Different Infectious Bovine Rhinotracheitis Marker Vaccines in Calves. Vaccines.

[22] (2018). Commission Delegated Regulation (EU) 2018/1629 of 25 July 2018, Amending the List of Diseases Set out in Annex II to Regulation (EU) 2016/429 of the European Parliament and of the Council on Transmissible Animal Diseases and Amending and Repealing Certain Acts in the Area of Animal Health (Animal Health Law). Off. J. Eur. Union.

[23] (2018). Commission Implementing Regulation (EU) 2018/1882 of 3 December 2018 on the Application of Certain Disease Prevention and Control Rules to Categories of Listed Diseases and Establishing a List of Species and Groups of Species Posing a Considerable Risk for the Spread of These Diseases. Off. J. Eur. Union.

[24] (2018). Manual of Diagnostic Tests and Vaccines for Terrestrial Animals. https://www.woah.org/fileadmin/Home/eng/Health_standards/tahm/3.04.11_IBR_IPV.pdf.

[25] Carter G.R., Cole J. (1990). Diagnostic Procedure in Veterinary Bacteriology and Mycology.

[26] Li H., Karney G., O’Toole D., Crawford T.M. (2008). Long distance spread of malignant catarrhal fever virus from feedlot lambs to ranch bison. Can. Vet. J..

[27] Martucciello A., Balestrieri A., Righi C., Cappelli G., Scoccia E., Grassi C., Brandi S., Rossi E., Galiero G., Gioia D. (2023). Evaluation of an immunization protocol using bovine alphaherpesvirus 1 gE-deleted marker vaccines against Bubaline alphaherpesvirus 1 in water buffaloes. Vaccines.

[28] Righi C., Iscaro C., Ferroni L., Rosati S., Pellegrini C., Nogarol C., Rossi E., Dettori A., Feliziani F., Petrini S. (2022). Validation of a commercial indirect ELISA kit for the detection of Bovine alphaherpesvirus 1 (BoHV-1)-specific glycoprotein E antibodies in bulk milk samples of Dairy cows. Vet. Sci..

[29] Munch R. (2003). Robert Koch. Microbes Infect..

[30] Romera S.A., Perez R., Marandino A., LuciaTau R., Campos F., Roehe P.M., Thiry E., Maidana S.S. (2022). Whole-genome analysis of natural interspecific recombinant between bovine alphaherpesviruses 1 and 5. Virus Res..

[31] Thiry J., Keuser V., Muylkens B., Meurens F., Gogev S., Vanderplasschen A., Thiry E. (2006). Ruminant alphaherpesviruses related to bovine herpesvirus 1. Vet. Res..

[32] Thiry E., Muylkens B., Meurens F., Gogev S., Thiry J., Vanderplasschen A., Schynts F. (2006). Recombination in the alphaherpesvirus bovine herpesvirus 1. Vet. Microb..

[33] Thiry E., Meurens F., Muylkens B., McVoy M., Gogev S., Thiry J., Vanderplasschen A., Epstein A., Keil G., Schynts F. (2005). Recombination in alphaherpesviruses. Rev. Med. Virol..

[34] Schynts F., Meurens F., Detry B., Vanderplasschen A., Thiry E. (2003). Rise and survival of Bovine Herpesvirus 1 recombinants after Primary Infection and Reactivation from Latency. J. Gen. Virol..

[35] Bo Z., Li X. (2022). A Review of Pseudorabies virus variants: Genetics, vaccination, transmission, and zoonotic potential. Viruses.

